# High-sensitive cardiac Troponin T is superior to echocardiography in predicting 1-year mortality in patients with SIRS and shock in intensive care

**DOI:** 10.1186/1471-2253-12-25

**Published:** 2012-09-24

**Authors:** Lill Bergenzaun, Hans Öhlin, Petri Gudmundsson, Joachim Düring, Ronnie Willenheimer, Michelle S Chew

**Affiliations:** 1 Department of Anaesthesiology and Intensive Care, Institution of Clinical Sciences, Skåne University Hospital, Lund University, Inga Marie Nilssons gata 47, S-20502 , Malmö, Sweden; 2 Department of Cardiology, Institution of Clinical Sciences, Skåne University Hospital, Lund University, Getingevägen 4, S- 22185 , Lund, Sweden; 3 Department of Biomedical Science, Malmö University, Södra Förstadsgatan 101, S- 20506 , Malmö, Sweden; 4 Department of Anaesthesiology and Intensive Care, Institution of Clinical Sciences, Skåne University Hospital, Lund University, Inga Marie Nilssons gata 47, S-20502 , Malmö, Sweden; 5 Heart Health Group, Lund University, Geijersg. 4C, 21618 , Limhamn, Sweden; 6 Department of Anaesthesiology and Intensive Care, Institution of Clinical Sciences, Skåne University Hospital, Lund University, Inga Marie Nilssons gata 47, S-20502 , Malmö, Sweden

**Keywords:** Echocardiography, BNP, High-sensitive TNT, Myocardial function, Mortality, Shock

## Abstract

**Background:**

Left ventricular (LV) dysfunction is well documented in the critically ill. We assessed 1-year mortality in relation to cardiac biomarkers and LV function parameters by echocardiography in patients with shock.

**Methods:**

A prospective, observational, cohort study of 49 patients. B-natriuretic peptide (BNP), high-sensitive troponin T (hsTNT) and transthoracic echocardiography (TTE) were assessed within 12 h of study inclusion. LV systolic function was measured by ejection fraction (LVEF), mean atrioventricular plane displacement (AVPDm), peak systolic tissue Doppler velocity imaging (TDIs) and velocity time integral in the LV outflow tract (LVOT VTI). LV diastolic function was evaluated by transmitral pulsed Doppler (E, A, E/A, E-deceleration time), tissue Doppler indices (é, á, E/é) and left atrial volume (La volume). APACHE II (Acute Physiology and Chronic Health Evaluation) and SOFA (Sequential Organ Failure Assessment) scores were calculated.

**Results:**

hsTNT was significantly higher in non-survivors than in survivors (60 [17.0-99.5] vs 168 [89.8-358] ng/l, p = 0.003). Other univariate predictors of mortality were APACHE II (p = 0.009), E/é (p = 0.023), SOFA (p = 0.024) and age (p = 0.031). Survivors and non-survivors did not differ regarding BNP (p = 0.26) or any LV systolic function parameter (LVEF p = 0.87, AVPDm p = 0.087, TDIs p = 0.93, LVOT VTI p = 0.18). Multivariable logistic regression analysis identified hsTNT (p = 0.010) as the only independent predictor of 1-year mortality; adjusted odds ratio 2.0 (95% CI 1.2- 3.5).

**Conclusions:**

hsTNT was the only independent predictor of 1-year mortality in patients with shock. Neither BNP nor echocardiographic parameters had an independent prognostic value. Further studies are needed to establish the clinical significance of elevated hsTNT in patients in shock.

## Background

Myocardial depression is a well-known complication of septic shock [1,2]. Raised levels of cardiac biomarkers such as natriuretic peptides [3,4] and cardiac troponin (cTn) [5], as well as echocardiographic changes of LV function [6-8] are frequently described. cTn is highly useful for both diagnosis and prognostication in patients with cardiac disease [5,9]. The recent introduction of a new generation of high sensitivity assays for cTn with lower cut-off values suggests advantages over traditional cTn in terms of accuracy [10], diagnosis [11] and prognostication [11,12] in patients with cardiac disease as well as in the general population [13]. In intensive care patients elevated cTN is related to mortality [14-16] and one study showed that hsTNT correlated to the severity of disease and was significantly higher in hospital non- survivors compared to survivors [17].

Elevated levels of natriuretic peptides such as B-natriuretic peptide (BNP) and amino- terminal fragment of BNP (NT-proBNP) are known to be strong prognostic markers in patients with cardiovascular disease [18,19]. In the critically ill raised levels of BNP and NT-proBNP can be found in many patients for a variety of reasons [20] and can be used as prognostic indicators [21,22]. Echocardiography is regarded useful for assessing cardiac function [23] but there are conflicting data regarding the prognostic value of LV systolic and diastolic function in patients in the intensive care unit (ICU) [3,4,24,25]. We investigated well established parameters of LV systolic [26-29] and diastolic function [30], where the latter have gained interest in ICU populations during the recent years [3,25].

The aim of this study was to investigate whether hsTNT, BNP and echocardiographic parameters of LV function measured within 12 h are associated with 1-year mortality in patients with shock.

## Methods

The study was approved by the Regional Ethics Review Board, Lund, Sweden (Dnr.187/2005). Informed consent was sought from the patient or, if not possible, from the next of kin. The study design was a prospective observational cohort study. Patients >18 years old admitted to the mixed-bed ICU of Skåne University Hospital, Malmö, Sweden, were screened for eligibility. We included 55 consecutive patients with Systemic Inflammatory Response Syndrome (SIRS) and shock, defined as failure to maintain mean arterial pressure ≥ 70 mmHg, despite adequate fluid resuscitation according to the surviving sepsis campaign algorithm [31]. Exclusion criteria were pregnancy, pre-existing abnormalities of coagulation, fibrinolytic therapy, compromised immunity or a “Do Not Attempt Resuscitation” order. Patients could only be included once. This study was part of a larger project over a 7 day period investigating other aspects of critical illness [32] independent of our study aim. APACHE II scores [33] were calculated at admission and SOFA scores [34] were calculated after 24 h. After the initial resuscitation period, fluids were given at the treating clinician’s discretion.

### Biochemical analyses

Blood samples were taken from an indwelling arterial line within 12 h of inclusion. They were sent to the local clinical chemistry laboratory, Skåne University Hospital, Malmö, Sweden, where they were centrifuged, frozen at −80°C and stored. hsTNT was measured using immunoassay (Cobas e601, Roche Diagnostics GmbH, Penzberg, Germany) [10]. The measuring range is 3–10 000 ng/L and the upper reference limit (99^th^ percentile) is 14 ng/l in healthy volunteers. The inter-assay coefficient of variation (CV) was <10%. Plasma BNP levels were analysed using UniCel^TM^ DxI 800 Beckman Access ® Immunoassay System (Beckman-Coulter Chaska, Brea, U.S.A.). The measuring range is 0.29- 1445 pmol/l and the upper reference limit is 30 pmol/l. The inter-assay CV was <10%. Biochemical samples were coded before analysis and laboratory personnel were blinded to clinical and echocardiographic data.

### Transthoracic echocardiography (TTE)

TTE examinations were performed within 12 h hours of inclusion by either of four experienced echocardiographers (LB, MC, PG, MD). Images were acquired using a Hewlett- Packard Sonos 5500 (Andover, Mass, U.S.A) scanner and a 3 MHz transducer. Two-dimensional (2D) imaging examinations were performed in the standard apical four- and two- chamber views (2C- and 4C views). Tissue harmonic imaging was used to enhance 2D image quality. Parameters of LV systolic function (LV ejection fraction [LVEF], mean atrioventricular plane displacement [AVPDm], peak systolic tissue Doppler velocity imaging [TDIs] and velocity time integral in the LV outflow tract [LVOT VTI]) were acquired as described previously [35]. Transmitral velocities were measured with pulsed-wave Doppler (PW) in the 4C view. For LV diastolic function, we used La volume and from the mitral inflow profile, the E- and A- velocity and E-deceleration time was measured. PW tissue Doppler recorded the diastolic velocities (é, á) of the LV septal wall at the level of the mitral annulus in the apical 4C view [36]. The E/A ratio as well as the E/é ratio, an index of LV filling pressure, were calculated [30]. La volume was estimated in the 4C view [37] and indexed to body surface area [30]. All TTE studies were recorded over three consecutive cardiac cycles independently of the respiratory cycle and averaged. In patients with non-sinus rhythm measurements were collected over 5–10 heartbeats. Analyses of the measurements were made >16 weeks after the data acquisition when the reader was less aware of the diagnosis in Phillip’s digital storing program Xcelera (Best, the Netherlands) offline.

### Statistical analysis

Data are presented as median (inter-quartile range [IQR]), percentages or absolute values. For not normally distributed variables we used non-parametric test exclusively. For correlation between two variables, Spearman’s rank correlation was used and for differences between two groups we used Mann-Whitney’s *U*-test. Categorical data were analyzed with Fisher’s exact test. HsTNT and BNP were log transformed with natural logarithm due to skewed distribution. Receiver operating characteristics (ROC) were used to define optimal cut-off values using the maximal area under the curve (AUC). Our aim was to investigate how 1-year mortality can be predicted by more than one explanatory variable measured early during ICU stay. Since we did not have any censored data during this period and odds ratio was the outcome of interest, logistic regression was chosen to be the most suitable method [38,39]. Multivariate (backward stepwise selection method with probability for the removal of 0.10) logistic regression analyses were used to determine the association of variables with 1-year mortality. Factors predictive of 1-year survival in univariate analyses were hsTNT, APACHE II, SOFA, E/é and age. As APACHE II score, but not SOFA score, is a validated general risk-prognostication system we used it in our logistic regression [40]. Additionally we performed a multivariate logistic regression with creatinine, as a marker of renal dysfunction, and pre-existing cardiac disease including atrial fibrillation. Odds ratios (OR) were calculated. The relationship between hsTNT quartiles and mortality was investigated by logistic regression. The intra- and interobserver variability of echocardiographic parameters was measured by the CV. CV was defined as the ratio of the standard deviation to the mean multiplied by 100. All probability values are two-tailed and significance was set at p < 0.05. The analyses were performed using SPSS 18.0 (SPSS, Chicago, IL, U.S.).

## Results

The original study included 55 consecutive patients. Two patients were excluded due to lack of written consent. One patient died 4 h after study inclusion and before echocardiographic examination, one patient was too obese to allow TTE and one patient was incorrectly registered in the echocardiography database. One patient moved abroad after 6 months, which precluded longer-term follow-up. These six patients were excluded from statistical analysis, resulting in a total of 49 analysed patients*.* Two-thirds of the population suffered from septic shock. The remaining patients suffered from shock due to other causes (pancreatitis, post-major non-cardiac surgery, intoxication and multiorgan failure, gastrointestinal bleeding and portal hypertension or unknown cause). Pre-existing cardiac disease was present in 24% of patients, defined as severe arrhythmia, heart failure or ischemic heart disease. Norepinephrine was used as a vasopressor. Twelve patients received dobutamine and one adrenaline at inclusion. Ten patients received levosimendan during the study period. In all, 49% had pre-existing treatment with β-blockers, ACE-inhibitors, Ca-channel blockers, and/or nitrates.

### Biochemical cardiac markers

**Figure 1:**
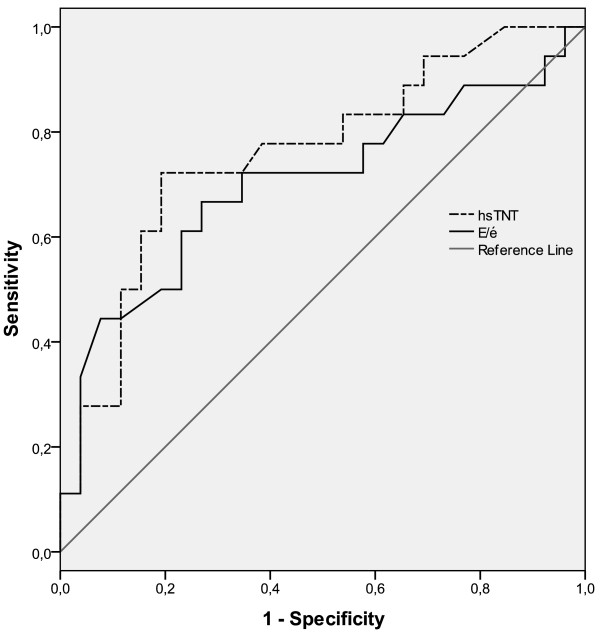
**Receiver operating characteristic (ROC) for hsTNT and E/é.** With regards to 1-year mortality the area under the curve (AUC) for high-sensitive Troponin T (hsTNT) was 0.76 (95% CI 0.612- 0.907, p = 0.004) and for E/é 0.703 (95% CI 0.535- 0.871, p = 0.023).

HsTNT was detectable in all 49 patients, ranged from <5 to 2592 ng/l (median 80 ng/l [IQR 24.0-193.5]) and was elevated (>14 ng/l) in 45 (92%) patients. With regard to 1-year mortality, AUC for hsTNT was 0.76 (95% CI 0.612- 0.907, p = 0.004), with 72% sensitivity and 82% specificity for a cut-off value of 117.5 ng/l (Figure 1). BNP ranged from 29 to 2031 pmol/l (median189 pmol/l [IQR 107–375]) (Table 1) and was elevated (>30 pmol/l) in 48 (98%) patients. AUC for BNP was 0.603 (95% CI 0.415 to 0.791, p = 0.26). hsTNT correlated with critical illness scores APACHE II [r = 0.335, p = 0.019] and SOFA [r = 0.301, p = 0.036]. There was no significant association with BNP, age, gender, diabetes, previous cardiac disease, E/é, lactate levels or creatinine.

**Table 1:** Patient characteristics

**Variable**	**n = 49**
**Demographics**	
Median age, y	65 (54–74)
Female sex	14 (29%)
**Previous medical history**	
Diabetes mellitus	6 (12%)
Hypertension	12 (24%)
Cardiac disease	12 (24%)
Pre- existing therapy	24 (49%)
**Clinical data**	
APACHE II	24 (19–29)
SOFA score	11 (9–13)
Mechanical ventilation,%	90
**Biochemical markers**	
Creatinine, μmol/l	155 (92.0-231.0)
Lactate, mmol/l	2.3 (1.6-3.3)
hsTNT, ng/l	80 (24.0-193.5)
BNP, pmol/l	189 (107–375)
**Echocardiographic data**	n = 46
LVEF,%	45 (40–55)
La volume, ml/m2	24 (21.0-31.6)
AVPDm, mm	10.7 (8.0-12.7)
E, cm/s	89 (71–104)
A, cm/s	67 (52–91)
E/A	1.3 (0.9-1.5)
DT, ms	165 (148–200)
TDIs, cm/s	8.6 (7.1-10.0)
é, cm/s	8.4 (7.1-10.0)
á, cm/s	9 (7.5- 12.3)
E/é	10.1 (8.5-12.2)
LVOT VTI, cm	17 (15–23)
**Mortality**	
7 day mortality,%	16
28 day mortality,%	27
1 year mortality,%	37
ICU mortality,%	27

Data are presented as median (lower quartile- upper quartile) or in numbers (%). APACHE II=Acute Physiology and Chronic Health Evaluation; SOFA=Sequential Organ Failure Assessment.

### Echocardiography

A total of 46 echocardiographic examinations were available for analysis, since 3 examinations were lost during the installation of a new offline storage and analysis system. The intra- and interobserver variability for echocardiographic parameters of LV systolic function ranged from 3.1% to 9.9% as reported earlier [35] and for echocardiographic parameters of LV diastolic function from 3.2% to 9.6%. There were no significant differences between survivors and non-survivors in any of the measured LV systolic function parameters (Table 2). The LV diastolic function parameters, E/é and La volume, surrogates of LV filling pressure, differed significantly between survivors and non-survivors (E/é median 9.9 vs 11.7, p = 0.023; La volume median 24 ml/m2 vs 31 ml/m2, p = 0.024) respectively (Table 2). In this study La volume was only feasible in 38 patients. Further, as La volume was less significant than E/é, E/é was chosen for further calculations. E/é correlated with age (r = 0.474, p = 0.001). There was no significant association between E/é and hsTNT, APACHE II, SOFA, lactate, BNP, La volume, gender, diabetes or previous cardiac disease. E/é was under 8 in 18%, between 8 and 15 in 71% and over 15 in 11% of patients. With regards to 1-year mortality, AUC for E/é was 0.703 (95% CI 0.535- 0.871, p = 0.023) with 72% sensitivity and 65% specificity for a cut-off value of 10.1 (Figure 1). The other LV diastolic function parameters did not differ significantly between survivors and non-survivors (Table 2).

**Table 2:** Patient characteristic according to 1-year survival

**Variable**	**Survivors (n = 31)**	**Non- survivors (n = 18)**	** p**
**Demographics**			
Median age, y	60 (49.5-68.5)	72 (68.3-76)	0.031
Female sex	10 (32%)	4 (22%)	ns
**Previous medical history**			
Diabetes mellitus	4 (13%)	2 (11%)	ns
Hypertension	9 (29%)	3 (17%)	ns
Cardiac disease	8 (26%)	4 (22%)	ns
Pre-existing therapy	14 (45%)	10 (56%)	ns
**Clinical data**			
APACHE II	22 (16–26)	28.5 (21–34)	0.009
SOFA score	10 (9–13)	13 (11–14)	0.024
Mechanical ventilation	29 (94%)	15 (83%)	ns
**Biochemical markers**			
Creatinine, μmol/l	154 (88–221)	171 (105–238)	ns
Lactate, mmol/l	2.2 (1.6-3.2)	2.5 (1.7-4.4)	ns
hsTNT, ng/l	60 (17.0-99.5)	168 (89.8-358)	0.003
BNP, pmol/l	159 (84–339)	241 (149–446)	0.26
**Echocardiographic data**			
	**Survivors (n = 28)**	**Non- survivors (n = 18)**	
LVEF,%	48 (40–55)	45 (36–65)	0.87
La volume, ml/m2	24 (20–27)	31 (25–36)	0.024
AVPD, mm	11.5 (8.8- 13)	9.0 (7.5-11)	0.087
E, cm/s	83.5 (68.8-96.3)	102.5 (75.5-113.0)	ns
A, cm/s	67.5 (57.0-92.3)	63.5 (40.0-86.0)	ns
E/A	1.2 (0.85-1.4)	1.4 (1.0-1.9)	ns
DT, ms	170 (150–200)	160 (135–195)	ns
TDIs, cm/s	8.5 (7.2-9.7)	8.7 (6.7-10)	ns
é, cm/s	8.4 (7.5-10.8)	7.9 (6.3-9.6)	ns
á, cm/s	9.9 (7.7-12.3)	8.8 (7.5-10)	ns
E/é	9.9 (8.1-10.9)	11.7 (9.8-14.8)	0.023
LVOT VTI, cm	20 (15–23)	17 (16–23)	0.18

Data are presented as median (lower quartile- upper quartile) or in numbers (%).

### Predictors of 1 year-mortality

Univariate analysis showed that hsTNT levels were significantly higher in non-survivors (median 168 [IQR 89.8-358] ng/l) than in survivors (median 60 [IQR 17–99.5] ng/l), p =0.003 while BNP was not significantly different (p = 0.26). Other predictors identified by univariate analysis were APACHE II, SOFA, age and the LV diastolic function parameters E/é and La volume (Table 2). Out of 45 patients with TNT values > 14 ng/l (99^th^ percentile), 18 (40%) were non-survivors and 27 (60%) were survivors. The remaining four patients with hsTNT ≤ 14 ng/l were all survivors.

A multivariable logistic regression analysis including hsTNT, APACHE II, E/é and age, identified hsTNT (p = 0.010) as the only independent predictor of 1-year mortality with an adjusted OR of 2.0 (95% CI 1.2- 3.5). Logistic regression showed increasing odds ratios for mortality for increasing hsTNT quartiles (OR of 3.7 [95% CI 0.3- 41.6], p = 0.294; OR of 9.4 [95% CI 0.93- 95.9], p = 0.058; OR of 22 [95% CI 2.1- 236.1], p = 0.011). When we included SOFA score to the model it became unstable due to correlations between the explanatory variable SOFA score and APACHE. When including creatinine and pre-existing cardiac disease (including atrial fibrillation) as independent variables in the model this did not affect the model or contribute with any significance.

## Discussion

The main findings of this study are: 1) hsTNT was the only independent predictor of 1-year mortality after adjustment for other factors; 2) elevated levels of hsTNT were found in the majority of patients; 3) E/é was higher in non-survivors; 4) neither BNP nor echocardiographic LV systolic function parameters were predictive of 1-year mortality.

### Early elevation of hsTNT is an independent predictor of 1-year mortality in critically ill patients with shock

Elevation of cTn is common among ICU patients for several reasons including myocardial infarction, sepsis and renal failure [41] and is known to be predictive of mortality during shorter follow-up periods such as ICU- and hospital mortality [14,15,41-44]. Even in critically ill patients where coronary artery disease has been excluded, elevated cTn is known to be associated with increased mortality [16]. In medical ICU patients, elevated cTn measured within 12 h of admission has been shown to be an independent risk factor for 30-day and 2-year mortality after adjustment for severity of illness [42]. The recent introduction of a new generation of high sensitivity assays for cTn, with a lower detection limit and sufficient analytical precision [9,10], allows the detection of elevated cTn in a considerably higher frequency compared to earlier essays (97% vs 76%) in patients with cardiovascular disease [10,11]. In general, renal insufficiency and cardiologically ill populations, detectable levels of hsTnT are associated with adverse outcomes [13,45,46]. However in critically ill patients, information about the value of high sensitive cTn is scarce. In a study by Rosjö et al. [17], hsTNT on inclusion was detectable in all patients with sepsis and septic shock. Further, hsTNT correlated to severity of disease and was significantly higher in hospital non-survivors but could not be identified as an independent predictor of mortality. Reynolds et al. [15] showed that an increased cTnI concentration while in ICU was associated with increased mortality in hospital, after adjusting for admission characteristics, age, severity of illness at admission, organ support, and serum creatinine concentrations. These results are in line with our study, where hsTNT measured within 12 h was detectable in 100%, elevated in 92% of patients, and correlated significantly with critical illness scores. Further, hsTNT was identified as the only independent predictor of even longer-term (1-year) mortality. The findings are strengthened by the increased odds ratios for mortality for increasing quartiles of hsTNT identified in this population although the wide CIs indicate that larger studies are needed to support our findings.

We also note that median hsTnT in non-survivors was higher in our study compared to the study by Rosjö et al.: 168 vs 54 ng/l. As the frequency of cardiovascular disease was comparable in both studies (24% and 26% respectively) we speculate that this might be due to sicker patients in our non-survivor group (median SOFA 13 vs 9 in Rosjö et al.). Since this was an exploratory study, and due to the paucity of literature regarding accepted levels of hsTNT in the critically ill, we chose the cut-off level with the best balance of specificity and sensitivity. We do not know if this is adequate and we hope that future studies will inform us as to what to expect from different critically ill populations. We note that the cut-off point identified by our ROC analysis is much higher than that identified in non-critically ill patients. The reason for this and its relevance is unclear and deserves attention in future studies.

We investigated if there was a confounding relationship between hsTNT and pre-existing cardiac disease or renal insufficiency but found no significant association which is propably due to sample size. Additionally when entering these confounders into the multivariable model, hsTNT could still be identified as the sole significant predictor of mortality.

### Diastolic but not systolic function parameters are associated with mortality

Impairment of myocardial function in patients with shock is often masked by concurrent elevations in cardiac index and a low systemic vascular resistance, making parameters such as LVEF often unreliable for monitoring LV systolic function and as a prognostic marker [1,47]. Additionally, markers of LV systolic function are frequently described to be normal or near normal [3,4,24] in these patients. This is in line with our results, where all echocardiographic parameters of LV systolic function were normal or mildly reduced and none were predictive of 1-year mortality. LV diastolic dysfunction with increased filling pressures is known to be predictive of mortality in cardiac patients [30] but has shown conflicting results regarding prognosis in ICU patients [3,4,24,25]. In our study E/é and La volume, both surrogates of LV filling pressure, were predictive of mortality but other diastolic parameters were not. E/é did, as expected, correlate significantly with age but not with hsTNT, BNP, APACHE II, SOFA score or lactate. These results were to some extent unexpected, as E/é has been shown in previous studies to be correlated to the severity of critical illness (3, 47).

Recent studies allude to the importance of E/é to prognosis in critically ill patients with shock [3,25]. Sturgess et al. [25] identified E/é as an independent predictor of hospital mortality, although with a considerably higher cut-off value than in our study (E/é = 14.5). This might be attributable to a higher percentage of pre-existing cardiac disease (43% in that study vs 24% in our study) and narrower inclusion criteria (septic shock vs shock). An É/é ratio < 8 cm/s is usually associated with normal filling pressure and E/é ratio > 15 cm/s commonly with increased filling pressure [30]. In our study median E/é was 10.1, thus representing a level between 8–15 where filling pressures might be elevated according to international guidelines [30]. Our study population represents a group of patients with increased age, pre-existing cardiovascular disease as well as acute critical illness. All can affect diastolic function and thus filling pressures. As we do not know to what extent E/é is affected by either of these different entities, we cannot separate their cardiac effects. Although E/é was only mildly increased it was still predictive irrespective of the underlying cause.

### BNP is not a valuable marker of 1-year mortality in this population

The role of natriuretic peptides as prognostic markers is well described in patients with cardiovascular disease [18]. Even in ICU patients several outcome studies refer to their usefulness [21,22]. Nevertheless the prognostic value of natriuretic peptides has been described as questionable [4], as they can be elevated due to a variety of reasons in critically ill patients [20]. Age, gender, pre-existing or critical illness associated renal and myocardial impairment as well as inflammatory states such as sepsis or septic shock all affect BNP [20,22]. In our study, elevated BNP was seen in most patients (98%) but did not correlate with critical illness (APACHE II) or organ dysfunction (SOFA), nor discriminated survivors from non-survivors. As LV systolic function overall was near normal and no patient had acute heart failure as the sole diagnosis, we speculate that elevated BNP due to other factors than heart failure is of little prognostic value.

### Limitations

Our study group containing the sickest of ICU patients with hemodynamic instability, implying cardiovascular impairment, is prone to bias and our results could have been completely different in another set of ICU patients. Therefore there is a risk of bias that could have led to overestimation of the prognostic ability of hsTNT. We have not excluded patients with known heart failure or atrial fibrillation, nor have we excluded patients with new onset of atrial fibrillation during their critical illness, which might have influenced our results. We did not record the absence or presence of ischemic ECG changes. This could have been of additional value to the echocardiographic examination in interpreting the likely cause of hsTNT elevations although this was not the aim of this study. In this observational study our intention was to investigate a group of critically ill patients with shock knowing that increased age, the likelihood of pre-existing cardiovascular disease and critical illness induced cardiac abnormalities such as atrial fibrillation, ischemic and/or cardiomyopathy probably would influence our results. Excluding patients with pre-existing cardiac disease would probably have reduced the cardiological impact of the regression model but would also have made the sample less representative of the population of critically ill patients. Since coronary angiography was not a possibility in this study, and since patients did not have pre-morbid echocardiograms, it is possible that we may have identified a subpopulation of critically ill patients with cardiac disease. We maintain however, that in this general group of very ill patients with shock, only hsTNT was indentified as a predictor of 1-year mortality, regardless of aetiology and background co-morbidity. A larger study stratifying BNP by age and gender might have yielded different results. TDI measurements were only done at the septal mitral annulus whereas current recommendations include both the septum and lateral wall [30]. Further a blinded assessment of echocardiographic data would have been desirable. Finally, the sample size is small; hence only limited variables could be used for the model, increasing the likelihood of confounding. We have attempted to reduce this by using univariate analysis to identify probable predictors, limiting the number of predictors and including these in the multivariate model. The results were congruent for different models that showed some consistency over the outcome predictor.

The strength of this study is that both LV systolic and diastolic echocardiographic measurements together with cardiac biomarkers were analysed as predictors of longer-term outcome.

## Conclusion

In this observational, cohort study, we found that hsTNT seems to be important for prognosis in the critically ill. Although in our study early measurement of hsTNT correlated with critical illness scores and was identified as the only independent predictor of 1-year mortality, clinicians should be aware that studies as ours are explorative and the results should be interpreted cautiously. Future studies should inform us on the reproducibility of these results, what levels to expect in different critical care subpopulations, what decision limits to implement and their clinical significance.

## Competing interests

The authors declare that they have no competing interests.

## Authors’ contributions

LB, RW, MC, PG, JD have made substantial contributions to conception and design of the study. LB, MC and HO participated in interpretation of data, helped to draft the manuscript. JD made contributions acquisition of data. PG, LB, MC made substantial contributions in acquisition and analysis of data. All authors have made substantial intellectual contributions to the manuscript and have given final approval of the version to be published. All authors read and approved the final manuscript.

## Pre-publication history

The pre-publication history for this paper can be accessed here:

http://www.biomedcentral.com/1471-2253/12/25/prepub
